# Mental healthcare in paediatric epilepsy clinics: implementation by non-mental health professionals

**DOI:** 10.1136/bmjpo-2024-002973

**Published:** 2024-11-18

**Authors:** Anna E Coughtrey, Sophie Bennett, Cameo Stanick, Bruce Chorpita, Emma Dalrymple, Peter Fonagy, J Helen Cross, Tamsin Ford, Isobel Heyman, Rona Moss-Morris, Poonam Jetha, Pamela Myles-Hooton, Roz Shafran

**Affiliations:** 1 UCL Great Ormond Street Institute of Child Health, London, UK; 2 Great Ormond Street Hospital for Children NHS Foundation Trust, London, UK; 3 King's College London Institute of Psychiatry Psychology & Neuroscience, London, UK; 4 Sycamores-Hathaway Centre for Excellence, Los Angeles, California, USA; 5 UCLA, Los Angeles, California, USA; 6 Division of Psychology and Language Sciences, UCL, London, UK; 7 Department of Psychiatry, University of Cambridge, Cambridge, UK; 8 Cambridge University Hospitals NHS Foundation Trust, Cambridge, UK

**Keywords:** Child Psychiatry, Psychology, Neurology

## Abstract

**Objectives:**

Research has shown that children with epilepsy often experience mental health disorders but face barriers to effective care. One solution is to train healthcare professionals within paediatric epilepsy services to deliver psychological interventions. The aim of this paper was to examine aspects of treatment integrity of the ‘Mental Health Interventions for Children with Epilepsy’ (MICE) treatment, a modular cognitive behavioural therapy intervention for anxiety, depression and behavioural difficulties in childhood epilepsy.

**Methods:**

The MICE treatment was delivered in paediatric epilepsy clinics by 21 healthcare professionals with limited mental health experience, supported by a comprehensive training and supervision package. Data from 2269 treatment sessions with 166 young people were analysed to examine adherence to the MICE protocol. Audio recordings from a randomly selected sample of 251 sessions were rated for therapist competence, of which 30 were independently rated by a second expert.

**Results:**

Therapists administered the MICE intervention with integrity and closely adhered to the established protocol. Any adaptations made were related to the sequence of delivery rather than changes to the content.

**Conclusions:**

The overall findings indicate that professionals in paediatric epilepsy clinics can be effectively trained and supported to administer evidence-based mental health interventions. Additional research is required to explore the link between integrity and clinical outcomes, as well as to determine the most effective methods for training and supervision. This is crucial for ensuring the successful implementation of mental health interventions for children with epilepsy and concurrent mental health needs.

**WHAT IS ALREADY KNOWN ON THIS TOPIC**

- Mental health problems in children and young people with epilepsy are frequently undetected and untreated, in part because physical and mental health services are not integrated.
- The ‘Mental Health Interventions for Children with Epilepsy’ (MICE) study has demonstrated that a modular cognitive behavioural intervention for common mental health problems for young people with epilepsy integrated into paediatric epilepsy clinics is clinically and cost-effective.
- However, little is known about whether the therapists in the study delivered the treatment with skill or fidelity to the MICE protocol. This is important, given that the therapists had limited or no prior experience in delivering psychological interventions.

**WHAT THIS STUDY ADDS**

- This study demonstrates that health professionals were able to deliver the MICE intervention with competence and with few adaptations to the flexible modular treatment protocols.

**HOW THIS STUDY MIGHT AFFECT RESEARCH, PRACTICE OR POLICY**

- With comprehensive training and suitable ongoing supervision, health professionals may be well placed to deliver psychological interventions such as MICE to improve access to mental healthcare from within paediatric settings.

## Introduction

 Children and young people with epilepsy (CYPE) are more likely to experience multiple common mental health conditions such as anxiety, depression and behavioural difficulties than youth without chronic physical health conditions.^1^,^3^ Despite this, the majority of mental health difficulties go undetected and untreated, leading to significant impairment and impact on quality of life into adulthood.^3^ The ‘Mental Health Interventions for Children with Epilepsy’ (MICE) study is a pioneering research study which co-created and evaluated a transdiagnostic cognitive behavioural therapy (CBT) intervention for managing anxiety, depression and behavioural difficulties in CYPE.^4 5^ The MICE intervention, based on the MATCH-ADTC framework^6^ is modular, enabling tailored treatments for more than one mental health problem within the specific context of childhood epilepsy.^4 5^ A large-scale multi-site randomised controlled trial found significant mental health improvements in CYPE aged 3–18 years who received the MICE intervention compared with standard care, with benefits sustained over 12 months.^7 8^

A novel feature of the MICE study was that the intervention was delivered by trained therapists from within epilepsy clinics, promoting integrated care. This model aimed to broaden access to evidence-based psychological interventions and to expand the ongoing availability and reach of MICE. While this model is beneficial for the long-term implementation of MICE, it also involved training health professionals who did not have prior training and expertise in delivering CBT.^9^ Therefore, it is important to understand how faithfully the MICE intervention was delivered, including adherence, fidelity and therapist skill.^7 9 10^

Previous published research into treatment integrity for MATCH-ADTC, on which MICE is based, has explored the discrepancies between what is expected to happen in a treatment session and what actually did happen, across multiple domains and types and levels of analyses.^11^ Determining what should happen in a psychological therapy session involves clinical decision-making drawing on various information sources including the evidence base, therapy components and activities outlines in treatment manuals, supervisor input and consideration of the patient’s response to the treatment to date.^11^ This framework facilitates a more nuanced understanding of integrity which focuses on the balance of inputs rather than an over-reliance on one information source. This is particularly relevant for modular, transdiagnostic psychological interventions such as MICE which have multiple dimensions of integrity. For instance, a therapist who rigidly continues to use a procedure that is recommended in a treatment manual despite evidence (eg, from weekly session-by-session measurement) that it is not working, would not be said to be implementing a treatment with integrity.^11^

Our previous work has demonstrated the clinical effectiveness of the MICE intervention on improving mental health symptoms and quality of life in CYPE over 12-month follow-up.^7 8^ The goal of this current study was to evaluate the implementation of the MICE intervention by trained and appropriately supervised therapists who had no prior experience of delivering CBT interventions. Specifically, we aimed to examine (1) the extent to which session content unfolded in an order and sequence consistent with the MICE treatment manual; and (2) therapist competence, the extensiveness and thoroughness of the chosen treatment element. This approach follows similar research exploring integrity in the implementation of MATCH-ADTC on which the MICE intervention is based.^11^

## Methods

Data collection occurred during the treatment phase of a randomised controlled, multi-centre trial of the clinical and cost-effectiveness of MICE between 2019 and 2022.^4 7^

### Patient and public involvement

Active involvement of CYPE and their caregivers was integral throughout the MICE research programme including this current study, from the funding application stage, through to project design, implementation, evaluation and dissemination. We recruited a diverse, committed and highly active research advisory group of 5 CYPE and 12 caregivers, and employed a patient and public involvement lead who is a coauthor on this paper (ED).

### Therapist participant sample

The MICE intervention was delivered by 21 health professionals from a range of backgrounds (consultant paediatricians n=2, paediatric nurses and epilepsy nurse specialists n=6, assistant psychologists n=9, trainee clinical psychologists n=2 and a junior doctor n=1). 19 of the 21 were female. The mean age was 30.94 years (SD=8.91; range=23–51), average years since qualification in core profession (n=9) was 12.44 years (SD=6.73; range=2–24) and mean years of experience of working with CYPE was 2.1 years (SD=4.24; range=0–13). The assistant psychologists (n=9) all had master level degrees in psychology so may have had theoretical knowledge of CBT but did not have prior practical experience in delivering CBT.

### Patient participant sample

CYPE aged 3–18 years were recruited from 13 epilepsy services across England and Northern Ireland. Out of the 334 participants in the study, 166 (85 male; M age=10.5 years; 122 White British) were randomly chosen to receive the MICE intervention. 93 (56%) had a primary diagnosis of disruptive behaviour on a standardised clinical interview (Developmental and Well-being Assessment),^12^ 66 (39.8%) had anxiety and 7 (4.2%) depression. Additionally, 40 (24.1%) had been diagnosed with autistic spectrum conditions, and 67 (40.4%) with intellectual disability.

### MICE intervention

The MICE intervention comprised up to 20 sessions delivered via telephone over 6 months, with an additional two booster sessions. Treatment included psychoeducation about mental health and epilepsy, CBT techniques for anxiety, depression and behavioural difficulties, and optional sessions on stigma, parental mental health and transition to adult services. The treatment was flexible, personalised and included epilepsy specific examples throughout.

To assess treatment integrity all sessions were recorded. Participants typically completed 16 sessions (IQR=12–19), resulting in a total of 2269 therapy sessions for analysis. All completed sessions were included in the assessments of integrity, regardless of the total number of sessions completed by the participant. All completed sessions were included in the assessments of integrity, regardless of the total number of sessions completed by the participant. There was no minimum number of sessions a participant had to attend to be classed as having received an adequate amount of the intervention. All participants had an assessment and at least one therapy session, with 158/166 (95%) having at least three sessions.

Therapists completed between 16 and 557 sessions of treatment (M=126.68 and SD=158.80). A single therapist delivered all the sessions for the patient, except when there were specific circumstances where treatment was transferred to a new therapist if the therapist’s job role changed prior to the patient completing treatment (eg, if a therapist embarked on a clinical psychology training programme). This occurred for 25/166 patients.

### Therapist training and supervision

Therapists underwent a rigorous 6-month training including 5.5 days of practical workshops, regular supervision and completion of a minimum of one training case. Training included CBT techniques, goal setting, therapeutic relationship and risk management. Supervision was provided by a clinical psychologist (AEC or SB) and focused on skill development and monitoring patient progress. With the exception of the assistant psychologists who received weekly hourly supervision, therapists received flexible supervision of variable frequency and length based on therapist and patient needs. For example, more regular supervision was triggered by a lack of patient progress. Full details of therapist training and supervision have been published elsewhere.^4 9^

### Therapist adherence

Therapist adherence to the MICE protocol was evaluated across all 2269 treatment sessions following previously established guidelines.^13 14^ The MICE protocol incorporates decision flowcharts to provide flexible treatment plans for each problem area (anxiety, low mood, behavioural problems). These flowcharts outline a standard order of treatment elements and include suggested flexible adaptations tailored to each patient’s unique presentation and needs. Previous research has developed adherence pairings to cover all possible pairings between the session of interest (‘index session’) and the previous session, allowing for sessions to be repeated and revisited if needed.^13 14^ We assessed therapist adherence to the MICE flowcharts using these existing adherence pairings which we expanded to include any additional content and sequencing from the MICE protocol.

Therapists recorded the specific treatment elements they implemented in each session and these were reviewed to compare the session pairings (index session-previous session) against the MICE flowcharts by an expert in the MICE protocol (AEC). The percentage of sessions where therapists adhered to both the content and sequencing of the MICE protocol was calculated. Given that the index session content was based on therapist self-report, the flowchart adherence pairing was also applied to the 10% of randomly selected therapy session audio recordings selected for competence rating to verify the data provided by therapists.

Each session was categorised into ‘all expected content’, ‘some expected content’ or ‘no expected content’. A session was classified as ‘all expected content’ if any part of the session topic aligned with the flowchart.^13 14^ For sessions categorised as ‘some expected content’, adaptations were identified as: (1) ‘sequencing’ that is, the session was either ahead or behind in the protocol; (2) ‘unexpected change in focus’ that is, content from the MICE protocol but not aligned with the current clinical focus; and (3) ‘expected change in focus’ that is, due to interference or comorbidity, as indicated in the flowcharts. Sessions were labelled as ‘no expected content’ when essential practice elements outlined in the flowcharts were omitted, or when sessions consisted solely of additional content not included in MICE.

### Therapist competence

The Cognitive Therapy Rating Scale Revised (CTS-R)^15^ is a gold standard tool for measuring therapeutic tactical competence in delivering CBT within a single treatment session with high internal consistency and variable inter-rater reliability.^16^,^20^ It was used in this study to measure the extent to which the therapist implemented the chosen session with skill and thoroughness. The CTS-R consists of 12 domains (five generic therapeutic skills and seven areas specific to CBT), each rated on a 6-point scale. We used the national standard for competence on this measure that is, minimum pass mark of 50% and all domains scoring minimum of 2.

A random sample of 251 out of the available 2269 sessions was chosen by an independent member of the clinical trials unit. These sessions were rated by a CBT accredited clinician trained in the use of the CTS-R (AEC and PJ). Each therapist had at least one session rated for competence and to verify the adherence rating (range 1–60 sessions; M*=*12.3; SD*=*17.4). 30 of the 251 recordings were independently rated by an expert not associated with the MICE research team (PM-H).

## Results

### Treatment plan adherence

The overall adherence rate for the 166 patients ranged from 64% to 100% (M=92%; SD=8.85%) with 64 patients receiving an adherence score of 100% (ie, all sessions matched the treatment plan). 17 of the 21 therapists (81%) all achieved 100% treatment plan adherence with at least one of their patients. Table 1 details the frequency of alignment between the content reported by therapists and that outlined in the MICE treatment plan across the 2269 sessions. A significant majority of sessions (91%) adhered to the expected content. The most common adaptation, observed in 123 (5%) sessions, involved expected changes in focus due interference (eg, parental mental health) or comorbidities. Sequencing adaptations, where a session was skipped or conducted ahead of schedule, occurred in 60 sessions (3%). Only 15 sessions (<1%) included protocol material unrelated to the current problem focus or interference. No instances of additional content outside the MICE protocol were detected in the therapist-reported content. However, 16 sessions (<1%) were categorised as having ‘no expected content’ because they missed an essential session in the flowchart, for instance missing the *wrap up* session at the end of treatment, prior to *booster* sessions.

**Table 1:** Frequency of agreement between therapist reported session content and MICE flowcharts across 2269 sessions

Agreement of recommendation with MICE flowcharts	Number of sessions	%
All expected content	2055	90.57
Some expected content	198	8.73
Nature of adaptation	n=198	
Sequencing	60	30.30
Expected change in focus	123	62.12
Unexpected change in focus	15	7.58
No expected content	16	0.70

MICEMental Health Interventions for Children with Epilepsy

Out of the 251 audio recordings rated for therapist competence, the flowchart adherence pairing agreed with the therapist reported content in 92% of cases. The primary reason for discrepancies was therapist recording errors when a session was categorised as a review session rather than a repetition of a previous session.

### Therapist competence

The total scores on the CTS-R for the therapy sessions ranged from 50% to 85% (M=60%, SD=6.77%). Impressively, all 251 audio recordings evaluated met the established threshold for therapist tactical competence indicating that the content of the sessions was of high quality and skilfully delivered. Of the 30 audio recordings that were double rated, all received a pass mark, with each item scoring over 2 and the total CTS-R score exceeding the minimum requirement of 50%. This resulted in a 100% agreement between the two raters.

## Discussion

MICE is a modular intervention aimed at addressing the high levels of comorbidity and complexity seen in CYPE and mental health problems.^5^ The findings from this study indicate that, following training and with appropriate ongoing supervision, professionals within epilepsy services effectively used the MICE intervention to treat mental health problems in CYPE. Our findings show that the vast majority of MICE sessions were delivered with high competence and integrity with minimal adaptation to treatment plans.

The analysis revealed that the vast majority of MICE sessions adhered to ‘all expected content’. Most adaptations involved an expected change in focus, with therapists incorporating additional sessions from the MICE protocol to better address comorbidities or interferences. The flexibility inherent in the modular design of MICE likely contributed to the high adherence observed, as therapists could tailor the treatment without needing to introduce external material. This approach aligns with findings from a community study of the MATCH-ADTC protocol, which showed that therapists often leverage the flexibility of a modular treatment design to handle comorbidity while still focusing on the primary issue.^21^ In our study, a smaller proportion of adaptations involved sequencing changes that is delivering content in a different order to the treatment plan. These findings echo those from previous research, where modifications in therapy were more frequently related to sequencing rather than content.^13 14^

The consistent levels of adherence could be attributed to both therapist factors and the nature of the MICE intervention. A recent review^22^ highlighted that experienced professionals might deliver treatments more idiosyncratically over time, relying more on clinical judgement than strictly following evidence-based protocols. The therapists in our study had limited or no prior experience in delivering psychological interventions and it is possible that this lack of experience may have led them to adhere more closely to the MICE protocol. It is also possible that the medical context meant that therapists had fewer alternative strategies to draw on and therefore were more likely to implement practice elements from the MICE manual.

A key element of the MICE intervention is session-by-session measurement, which is used to monitor patient progress and identify factors contributing to change.^23 24^ These regular reviews of patient progress may have guided therapists in deciding when to deviate from the protocol for necessary adaptations, especially in sequencing. The role of supervision in clinical decision-making within MICE is an area of particular importance, as previous studies have indicated that therapists often modify the MATCH-ADTC protocol following supervisor consultations.^14^

Therapist competence in the MICE trial was good, with all of the randomly selected session recordings scoring above the competence threshold on the CTS-R. This indicates that therapists were not only faithful to the MICE protocol but also implemented treatment practices with therapeutic skill and proficiency beyond training and throughout the research period.^9^ This is particularly noteworthy as therapists did not have prior experience in delivering CBT to CYPE. Future research should delve into how the nature and frequency of training and supervision affect competence, especially for professionals without a mental health background.

### Limitations

While our current findings suggest that the therapists in the MICE trial could competently administer the practice elements recommended by the flowcharts, we did not assess the process of how clinical decisions were made. This is an important area for future research as the MICE intervention encouraged therapists to decide what strategies to implement by combining information from the flowchart recommendations, the current status and trend of the patient’s presenting difficulties and progress towards goals as shown by standardised session-by-session measures, and supervisor input. Further research is warranted to explore how these factors interact in the context of MICE, particularly in relation to therapy adaptations and the influence of supervisory guidance. Given that 11 of the therapists were either assistant psychologists (n=9) or trainee clinical psychologists (n=2) and therefore may have had prior theoretical knowledge of the principles underpinning the MICE intervention, it is important for future research to evaluate the impact of the MICE training on therapists with no prior mental health knowledge and experience.

There was a high level of agreement between the therapist-reported content and the independent rater flowchart adherence keys for a 10% random sample of sessions, but there remains a possibility that therapists might not have accurately recorded any therapy deviations. Furthermore, we did not select the recordings to ensure that they were evenly distributed across patients or therapists. Future research would benefit from directly analysing the recorded sessions, rather than solely relying on therapist self-report. Additional studies are also necessary to understand how, when and why therapists might introduce extraneous content or significantly deviate from the MICE protocol. While this current study has shown a good level of therapist fidelity and competence, future research is needed to replicate the findings and compare the clinical outcomes between health professionals trained in the MICE intervention who have limited prior mental health experience, with those who have received traditional mental health training for example, clinical psychologists. The relationship between patient outcomes and treatment integrity is also an important focus for future research, particularly focusing on the impact of modification to standard flowchart procedures and supervision input when patients are not showing treatment gains.

## Conclusions

In summary, this study has shown that despite limited prior experience, health professionals can be trained and supervised to effectively deliver a modular CBT intervention for CYPE. With appropriate training and supervisory structures, this approach may improve access to evidence-based psychological therapies for this underserved population and could be used as a model for training professionals working in other paediatric settings. Gaining a deeper understanding of the factors contributing to sufficient therapist competence is crucial for guiding the implementation of MICE and other evidence-based mental health interventions in physical health settings.

## Data Availability

Data are available upon reasonable request.
