# Supplementary material for: Mental healthcare in paediatric epilepsy clinics: implementation by non-mental health professionals
Source: BMJ Paediatr Open. 2024 Nov 18;8(1):e002973. doi: 10.1136/bmjpo-2024-002973 (PMC11574424; doi:10.1136/bmjpo-2024-002973)
Supplement: Supplementary file 1 [file bmjpo-2024-002973.reviewer_comments.pdf]

PEER REVIEW HISTORY

BMJ Paediatrics Open publishes all reviews undertaken for accepted manuscripts. Reviewers are asked to complete a checklist review form and are provided with free text boxes to elaborate on their assessment. These free text comments are reproduced below.

ARTICLE DETAILS

**TITLE (PROVISIONAL)**	Mental Health Care in Paediatric Epilepsy Clinics: Implementation by Non-Mental Health Professionals
**AUTHORS**	Coughtrey, Anna; Bennett, Sophie; Stanick, Cameo; Chorpita, Bruce; Dalrymple, Emma; Fonagy, Peter; Cross, J; Ford, Tamsin; Heyman, Isobel; Moss-Morris, Rona; Jetha, Poonam; Myles-Hooton, Pamela; Shafran, Roz

VERSION 1 - REVIEW

**REVIEWER NAME**	***Daniel Lumsden***
**REVIEWER AFFILIATION**	Evelina London Children's Healthcare
**REVIEWER CONFLICT OF INTEREST**	
**DATE REVIEW RETURNED**	27-Aug-2024

**GENERAL COMMENTS**	Thank you for the opportunity to review a revision of this submission. The authors have acknowledged the major limitation of this presented work in that the impact of the intervention has not been measured - instead the focus has been on the fidelity of the implementation.

**REVIEWER NAME**	***Sarah Nevitt***
**REVIEWER AFFILIATION**	University of York, Centre for Reviews and Dissemination
**REVIEWER CONFLICT OF INTEREST**	
**DATE REVIEW RETURNED**	10-Sep-2024

**GENERAL COMMENTS**	This manuscript describes the therapist adherence and therapist competence related to the MICE intervention within a recently conducted RCT in children and young people with epilepsy (CYPE).<br><br>I should note that my main area of expertise is statistics, rather than subject expertise, so my review concentrates on the statistical methodology and results outlined within this manuscript.<br><br>Overall the adherence and competence assessments seem to be appropriately conducted and well reported. The therapist adherence and competence are impressive, as the authors say.<br><br>I have only a few minor suggestions and queries for clarity for the authors:<br><br>1) Therapist participant sample: I suggest that quoting the ranges for these measures (age, years since qualification and years since working with CYPE) would also be useful.

	2) MICE intervention: I understand that the intervention comprises up to 20 sessions (plus two booster sessions) and on average participant completed 16 sessions, IQR 12 to 19 sessions. But was there a was there a minimum number of sessions that a participant had to attend to be classed as having received an adequate amount of the intervention?<br>Or for integrity to be assessed? i.e. were all completed sessions included in the assessments of integrity, regardless of the total sessions completed by the participant?<br><br>3) I appreciate that the aim here is to assess adherence and competence across all therapists rather than to focus on any individual therapist, however, I suggest that it may be informative to provide some summary statistics on how many sessions each of the 21 therapists delivered and whether participants received sessions from a single therapist or multiple therapists (if the latter, how many). While I don't claim to have any subject specific expertise in this area, I would imagine that treatment fidelity could vary depending on whether a single therapist delivers all sessions for a participant or multiple therapists.<br><br>4) Therapist adherence: "the flowchart adherence pairing was also applied to 10% of randomly selected therapy session audio recordings to verify the data provided by therapists." Is this the same randomly selected sample (i.e. 251 sessions) described below for which therapist competence was rated by a AC or PJ?<br><br>And were the random sample(s) evenly distributed across the 21 different therapists? i.e. approx 10-12 sessions per therapist? And/or across the 166 participants i.e. 1-2 sessions per participant?<br><br>5) Results, treatment plan adherence. The number of patients for which all sessions matched the treatment plan (i.e. an adherence score of 100%) is reported, but does adherence really apply to the patients if they are the ones receiving the session?<br>My understanding is that therapist adherence is being assessed here so how many therapists received an adherence score of 100%?

VERSION 1 – AUTHOR RESPONSE

**Reviewer 1**	
**Comment**	**Response**
In recent years there has been increasing awareness of the mental health challenges facing children and young people with epilepsy and other neurological disorders. One barrier to addressing these needs are limitations to the availability of mental health professionals. The authors in this report have examined whether non-mental health professionals can be trained to deliver a mental health intervention. The report focuses on how "well" non-mental health individuals deliver the intervention (fidelity to	Thank you for your comments and highlighting the importance of our work. As stated, the aim of this paper was to focus on how non-mental health individuals deliver the intervention. Our previously published work has demonstrated that the MICE intervention is effective in terms of improving mental health and well-being in children and young people with epilepsy (CYPE):

intended intervention) – not how effective this is in terms of improving mental health and well-being.<br><br>The report is well written and structured.	Bennett SD, Cross JH, Chowdhury K, et al. Clinical effectiveness of the psychological therapy Mental Health Intervention for Children with Epilepsy in addition to usual care compared with assessment-enhanced usual care alone: a multicentre, randomised controlled clinical trial in the UK. Lancet 2024; https://doi.org/10.1016/S0140-6736(23)02791-5
Whilst the authors suggest that the group being trained have limited mental health experience, it is notable that 11/21 subjects delivering the program were assistant of trainee assistant clinical psychologist who depending upon time in role, may have a greater experience. They note the assistants lack clinical experience of CBT, but have MSc level education in psychology, which suggests at least a degree of prior exposure to the theoretical underpinnings of these techniques.	Thank you for raising this point. We now say in the methods on page 6: *"The assistant psychologists (n=9) all had master level degrees in psychology so may have had theoretical knowledge of CBT but did not have prior practical experience in delivering CBT."*<br><br>We have also added the following to the limitations section on page 12: *"Given that 11 of the therapists were either assistant psychologists (n=9) or trainee clinical psychologists (n=2) and therefore may have had prior theoretical knowledge of the principles underpinning the MICE intervention, it is important for future research to evaluate the impact of the MICE training on therapists with no prior mental health knowledge and experience."*
The authors have demonstrated a good level of fidelity and competence with regards to the delivery of the MICE intervention.	Thank you. This was our aim with this paper, which supplements the existing published research on the clinical and cost-effectiveness of the MICE intervention.
The study is limited in not measuring the "impact" of the intervention. The implication is that if the process is followed, the results will be the same, but I do believe this bears further examination. There is no comparator in terms of how these results compare to more traditionally trained mental health professionals. From the data presented, it's clear that the delivery of MICE has been "good" – but is this "good" enough?	The impact of the intervention is reported elsewhere in the Lancet paper. The questions on the replicability of the findings and how these results compare to more traditionally trained mental health professionals are relevant for future research. We have now stated on page 12: *"Whilst this current study has shown a good level of therapist fidelity and competence, future research is needed to replicate the findings and compare the clinical outcomes between health professionals trained in the MICE intervention who have limited prior mental health experience, with those who have received traditional mental health training e.g. clinical psychologists."*

There does not appear to be a clear “limitations” section to the discussion? I would encourage the authors to highlight study limitations a little more clearly	We have now added a sub-heading highlighting the limitations in the discussion on page 12.
